# Change Processes in Cognitive Therapy for Social Anxiety Disorder Delivered in Routine Clinical Practice

**DOI:** 10.32872/cpe.v2i2.2947

**Published:** 2020-06-30

**Authors:** Graham R. Thew, Anke Ehlers, Nick Grey, Jennifer Wild, Emma Warnock-Parkes, Rachelle L. Dawson, David M. Clark

**Affiliations:** a Department of Experimental Psychology, University of Oxford, Oxford, United Kingdom; b Oxford University Hospitals NHS Foundation Trust, Oxford, United Kingdom; c Oxford Health NHS Foundation Trust, Oxford, United Kingdom; d Institute of Psychiatry, Psychology and Neuroscience, King’s College London, London, United Kingdom; e National Institute for Health Research Mental Health Biomedical Research Centre, South London and Maudsley NHS Foundation Trust, London, United Kingdom; f Sussex Partnership NHS Foundation Trust, Worthing, United Kingdom; Philipps-University of Marburg, Marburg, Germany

**Keywords:** social anxiety, cognitive therapy, change processes, structural equation model, mediation

## Abstract

**Background:**

Most studies examining processes of change in psychological therapy for social anxiety disorder (SAD) have analysed data from randomised controlled trials in research settings.

**Method:**

To assess whether these findings are representative of routine clinical practice, we analysed audit data from two samples of patients who received Cognitive Therapy for SAD (total N = 271). Three process variables (self-focused attention, negative social cognitions, and depressed mood) were examined using multilevel structural equation models.

**Results:**

Significant indirect effects were observed for all three variables in both samples, with negative social cognitions showing the strongest percent mediation effect. ‘Reversed’ relationships, where social anxiety predicted subsequent process variable scores, were also supported.

**Conclusion:**

The findings suggest the processes of change in this treatment may be similar between research trials and routine care.

## Abstract (highlights)

- The three process variables examined showed significant indirect effects on subsequent social anxiety.
- There was evidence of a bidirectional relationship between process and outcome.
- Results are consistent with the theoretical model underpinning the treatment.
- The change processes of this treatment in routine practice may be similar to those found in research trials.

There is good evidence for the efficacy of psychological therapies in the treatment of mental health problems. However, there is a less clear understanding of the exact processes through which they operate. Further research on mechanisms of clinical improvement has been highlighted as a significant need in clinical psychology (Emmelkamp et al., 2014; Holmes et al., 2018; Kazdin, 2007). If we can determine which process variables are involved in producing clinical improvement, it may be possible to adapt therapies to place more emphasis on these, and to implement techniques that target them earlier in therapy, so as to increase the efficacy and efficiency of treatment.

In Social Anxiety Disorder (SAD), there is a small but growing body of literature exploring process-outcome relationships in psychological therapy. The choice of process variables to be assessed is generally derived from theoretical accounts of SAD, such as the cognitive model of Clark and Wells (1995), and the cognitive-behavioural model of Rapee and Heimberg (see Hope, Heimberg, & Turk, 2006; Rapee & Heimberg, 1997). The Clark and Wells model specifies several anxiety-maintaining factors that are potential predictors of clinical change. These include negative social anxiety-related cognitions, avoidance and safety behaviours, and self-focused, evaluative attention. The Rapee and Heimberg model also highlights hypervigilance, avoidance and attentional bias towards perceived threat as potential mechanisms of anxiety maintenance. Besides anxiety-maintaining factors, other variables such as working alliance, or measures of the degree of compliance with clinical techniques, could be examined.

Five studies, mostly focusing on cognitive-behavioural interventions (Boden et al., 2012; Calamaras, Tully, Tone, Price, & Anderson, 2015; Goldin et al., 2014; Gregory, Wong, Marker, & Peters, 2018; Hoffart, Borge, Sexton, Clark, & Wampold, 2012), have shown evidence that changes in negative cognitions and threat appraisals were associated with improvements in social anxiety, while two studies (Mörtberg, Hoffart, Boecking, & Clark, 2015; Niles et al., 2014) did not find evidence of an association between changing negative cognitions and outcome. Evidence of changes in self-focused attention being associated with clinical improvement was found in the study by Mörtberg et al. (2015), and in both individual and group Cognitive Therapy (CT) in a study by Hedman et al. (2013). Two studies showed support for avoidance of social situations as a predictor of outcome (Aderka, McLean, Huppert, Davidson, & Foa, 2013; Hedman et al., 2013), and the study of participants’ use of exposure and thought records (Hawley, Rector, & Laposa, 2016) also supported a predictive relationship for these factors. In contrast, the two studies analysing working alliance either did not find a mediation relationship (Calamaras et al., 2015) or found that the alliance-outcome relationship was itself mediated by cognitive factors (Hoffart et al., 2012). The one study investigating physiological anxiety symptoms did not find evidence of a predictive association with outcome (Aderka et al., 2013), while the one study examining depression found a weak effect (Moscovitch, Hofmann, Suvak, & In-Albon, 2005).

Although it is promising that mediation and other predictive effects in treatments for SAD are starting to emerge, there is a lack of consistency across the studies to date regarding which process variables, and which treatments, are examined. It is rare for two studies to examine the same process variables within the same treatment. In addition, the participant samples analysed in the studies are almost all drawn from randomised controlled trials (RCTs), meaning there is a lack of research using data from routine clinical practice. Datasets from such settings typically include a larger number of therapists, some therapists who are less experienced, and fewer participant selection criteria relative to RCTs. In the same way that effectiveness studies in routine clinical settings complement efficacy studies, in that they can test whether findings from controlled research settings apply in routine practice (Gunter & Whittal, 2010; Kettlewell, 2004; Weisz, Ng, & Bearman, 2014), it can be argued that for a predictor to be considered reliable, it should operate similarly regardless of setting. It is therefore important to examine process-outcome effects within data from routine clinical practice.

The present study therefore aimed to explore change processes during Cognitive Therapy for Social Anxiety Disorder (CT-SAD) based on the Clark and Wells (1995) model delivered in routine clinical practice, using data from an audit of clinical outcomes from a specialist National Health Service (NHS) anxiety clinic in London. To be consistent with previous literature, negative social cognitions and self-focused attention were examined as process variables, and were measured in the same way as in previous studies (e.g. Hedman et al., 2013; Mörtberg et al., 2015). These variables have a strong theoretical basis given their key roles within the Clark and Wells (1995) model. In addition, depressed mood, which is not a component of the cognitive model of SAD, was investigated as an additional process variable to examine the specificity of any effects found using the other two theoretically-derived factors (see Preacher, 2015). There is, however, a plausible rationale for changes in depressed mood being associated with improvements in social anxiety, in that a reduction in depressed mood over time may be accompanied by greater hopefulness and optimism about treatment and the future, leading to subsequent improvement in social anxiety outcomes.

## Method

### Participants

Data were drawn from an audit of clinical outcomes of psychological therapy for SAD, which examined consecutive referrals to the Centre for Anxiety Disorders and Trauma, a UK NHS specialist clinic in London. The service receives referrals from general practitioners and community mental health teams. Assessments were completed between May 2001 and August 2010. All assessments were conducted by a trained clinician and included the Structured Clinical Interview for DSM-IV (SCID-IV; First, Spitzer, Gibbon, & Williams, 2002) to determine primary and comorbid diagnoses. The personality disorder screener questions of the SCID-II (First, Gibbon, Spitzer, Williams, & Benjamin, 1997) were also given, with further assessment undertaken as clinically indicated. All participants met DSM-IV criteria for SAD, with SAD being judged to be the main problem by the assessing clinician. Exclusion criteria were current psychosis, or dependence on alcohol or substances.

Across the audit period, 317 people were treated with CT-SAD. Three of these people were re-referred during the audit period and received a second course of treatment; only their first course of treatment was included in the analysis. Files of seven people who received treatment were not available for data entry. To be included in the present studies, participants were required to have attended at least five treatment sessions and completed the weekly questionnaires on at least five occasions. This ensured a sufficient number of measurement points per participant to permit analysis of process variables over time. As 23 participants attended fewer than five sessions, and 13 completed insufficient questionnaire data for analysis, the final sample size for the analysis of standard CT-SAD was 271.

These participants completed an average of 12.3 sessions (*SD* = 2.9). Six participants (2%) had more than 18 sessions and the greatest number of sessions attended was 26. Treatment extended over an average of 204.3 days (*SD* = 103.7). There were 69 participants who received their treatment as part of research trials running at the time.

Some of the outcome measures used by the clinic were changed in September 2008 when the clinic joined the Improving Access to Psychological Therapies (IAPT) programme (see Clark, 2018). Participants treated before (Sample 1; *n* = 185) and after (Sample 2; *n* = 86) this change in outcome measures were analysed separately. Demographic and clinical characteristics of both samples are shown in Table 1. The audit was approved by the local ethics committee.

**Table 1:** Demographic and Clinical Characteristics

Participant Variable	Sample 1 (*n* = 185)	Sample 2 (*n* = 86)	Total (*N* = 271)
**% Female**	48	52	49
**Mean age (*SD*)**	32.2 (8.6)	33.2 (9.5)	32.5 (8.9)
Marital status *n* (%)			
Married	19 (10.3)	22 (25.6)	41 (15.1)
Cohabiting	28 (15.1)	8 (9.3)	36 (13.3)
Widowed	1 (0.5)	0	1 (0.4)
Divorced	3 (1.6)	0	3 (1.1)
Separated	5 (2.7)	3 (3.5)	8 (3.0)
Single/Never married	120 (64.9)	45 (52.3)	165 (60.9)
Not given	9 (4.9)	8 (9.3)	17 (6.3)
Ethnicity *n* (%)			
Black	11 (5.9)	8 (9.3)	19 (7.0)
Caucasian	140 (75.7)	37 (43.0)	177 (65.3)
Indian	2 (1.1)	2 (2.3)	4 (1.5)
Pacific Asian	1 (0.5)	0	1 (0.4)
Other	6 (3.2)	0	6 (2.2)
Not given	25 (13.5)	39 (45.3)	64 (23.6)
Highest qualification *n* (%)			
Doctoral degree	7 (3.8)	2 (2.3)	9 (3.3)
Masters degree	18 (9.7)	11 (12.8)	29 (10.7)
Professional qualification	15 (8.1)	5 (5.8)	20 (7.4)
Bachelors degree	71 (38.4)	27 (31.4)	98 (36.2)
A Levels	31 (16.8)	12 (14.0)	43 (15.9)
GCSEs	23 (12.4)	9 (10.5)	32 (11.8)
None	12 (6.5)	3 (3.5)	15 (5.5)
Other	7 (3.8)	3 (3.5)	10 (3.7)
Not given	1 (0.5)	14 (16.3)	15 (5.5)
Employment status *n* (%)			
Unemployed	33 (17.8)	11 (12.8)	44 (16.2)
Full time	103 (55.7)	52 (60.5)	155 (57.2)
Part time	20 (10.8)	6 (7.0)	26 (9.6)
Self-employed	4 (2.2)	5 (5.8)	9 (3.3)
Sick leave	3 (1.6)	1 (1.2)	4 (1.5)
Retired	0	2 (2.3)	2 (0.7)
Student	17 (9.2)	2 (2.3)	19 (7.0)
Homemaker	2 (1.1)	1 (1.2)	3 (1.1)
Freelance	0	1 (1.2)	1 (0.4)
Compassionate leave	0	1 (1.2)	1(0.4)
Not given	3 (1.6)	4 (4.7)	7 (2.6)
**Mean age of SAD onset in years (*SD*)**	19.3 (8.4)	19.1 (7.1)	19.3 (8.0)
**Mean duration of SAD in years at assessment (*SD*)**	12.9 (9.5)	13.9 (10.8)	13.2 (9.9)
**% Prescribed psychotropic medication**	30	25	29

### Treatment

All participants received individual CT-SAD as described in Clark et al. (2006). Manuals, videos of workshops, and other therapist support materials are available at https://oxcadatresources.com (Oxford Centre for Anxiety Disorders and Trauma, 2019). The standard structure of treatment used in RCTs comprises 14 weekly sessions, followed by up to three booster sessions at monthly intervals. For the present participants treated in routine clinical practice, this structure was followed in most cases, but for some, adjustments in the number and spacing of sessions were made due to clinical need. End of treatment outcomes were taken from the last attended session.

### Therapists

Therapists were mental health professionals with a range of professional backgrounds including clinical psychology, counselling psychology, nursing and/or specialist CBT training. Some of the therapists were on training placements within the service (trainee clinical psychologists, trainee high intensity therapists, and specialist psychiatry registrars). A total of 22 therapists treated the participants in Sample 1, and 36 therapists for the participants in Sample 2. The number of participants seen by each therapist ranged from 1 to 24.

### Session-By-Session Measures

#### Self-Focused Attention

This was measured using the mean score of the two self-focused attention items in the Social Phobia Weekly Summary Scale (SPWSS; Clark, 1995, available at https://oxcadatresources.com) where people provide a rating of their self-focused attention in general, and in situations they found difficult, over the past week. The full six-item scale also elicits ratings of avoidance, anticipatory worry, and post-event rumination over the previous week, along with an overall rating of social anxiety. All items are rated on 0-8 Likert scales, with total scores ranging between 0 and 48. The SPWSS has been shown to be sensitive to treatment effects and has good internal consistency (Clark et al., 2006; Clark et al., 2003). Cronbach’s alpha in the present sample for the two self-focused attention items was .75 at baseline and .89 at end of treatment.

#### Negative Social Cognitions

The Social Cognitions Questionnaire (SCQ; Oxford Centre for Anxiety Disorders and Trauma, 2019; Wells, Stopa, & Clark, 1993) was used, which presents 22 negative social cognitions, each of which is rated for both the frequency with which it occurred in the last week when the respondent was anxious (rated from 1 = “thought never occurs” to 5 = “thought always occurs when I am nervous”), and the degree to which they believe the thought to be true when it occurs (rated from 0 = “I do not believe this thought”, to 100 = “I am completely convinced this thought is true”). Mean scores are calculated for Frequency (range 1-5) and Belief (range 0-100) with higher scores indicating more negative social cognition. Cronbach’s alpha in the present sample was .90 (baseline) and .96 (end of treatment) for the frequency subscale and .91 (baseline) and .97 (end of treatment) for the belief subscale. For the present studies the frequency and belief subscales were standardised and averaged to produce a single composite *z* score.

#### Depressed Mood

For Sample 1, depressed mood was measured using the Beck Depression Inventory (BDI; Beck & Steer, 1993). Cronbach’s alpha in the present sample was .91 at baseline and .94 at end of treatment. For Sample 2, depressed mood was measured using the Patient Health Questionnaire – 9-item version (PHQ; Kroenke, Spitzer, & Williams, 2001). Cronbach’s alpha in the present sample was .88 at baseline and .92 at end of treatment.

#### Social Anxiety

For Sample 1, social anxiety was measured using the Social Phobia Weekly Summary Scale (Clark et al., 2003; Oxford Centre for Anxiety Disorders and Trauma, 2019), minus the two attention items. A total social anxiety severity score was computed from the items: overall rating of social anxiety, avoidance, anticipatory worry, and post-event rumination. Cronbach’s alpha for the baseline and end of treatment scores were .74 and .91 respectively. For Sample 2, social anxiety was measured using the Social Phobia Inventory (SPIN; Connor et al., 2000), a 17-item scale listing a range of SAD-related problems, incorporating fear, avoidance, and physical symptoms. Cronbach’s alpha for the baseline and end of treatment scores were .90 and .93 respectively.

### Analysis

**Figure 1:**
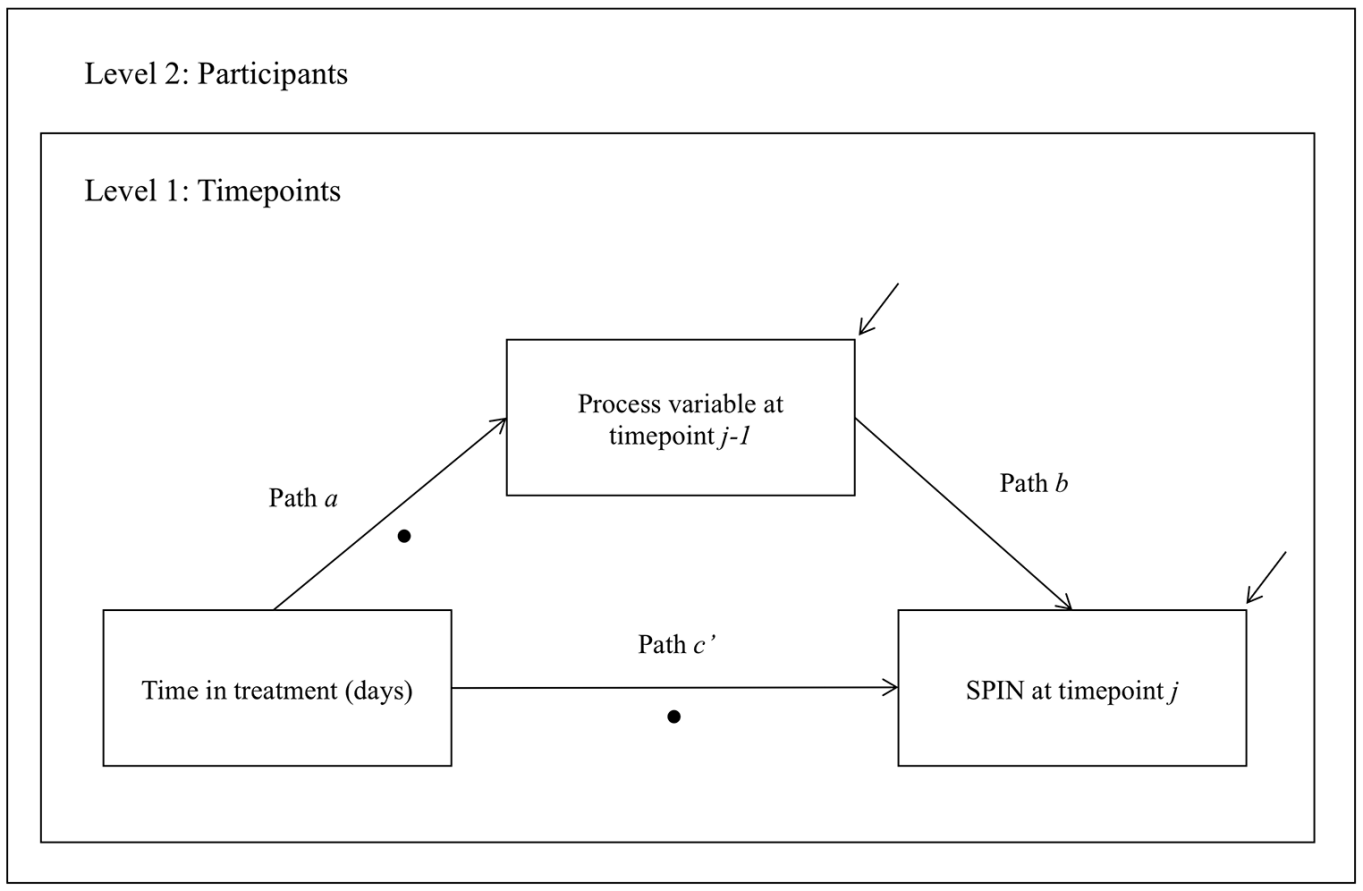
Simplified Path Diagram of Multilevel Structural Equation Model (MSEM) to Test the Indirect Effect of Time on Scores on the Social Phobia Inventory (SPIN) Via One of Three Process Variables *Note.* Filled circles indicate paths specified as random, and raised arrows indicate residuals.

A series of multilevel structural equation models (MSEM) were computed (see Preacher, Zhang, & Zyphur, 2011; Preacher, Zyphur, & Zhang, 2010) based on the analytic strategy of Mörtberg et al. (2015), with total scores at each session (Level 1) nested within participants (Level 2). Therapist was not included as a third level given the limited number of therapists^1^1Maas and Hox (2005) suggest group sizes over 50 at the higher level of multilevel models are most appropriate to avoid biased estimates., and the variability in the number of participants seen by each therapist. For two-level models, data simulations have shown that sample sizes of 50 and above produce unbiased parameter estimates under a range of conditions (Hox, Maas, & Brinkhuis, 2010). The number of elapsed days in treatment was used as the independent variable, and severity of social anxiety as the dependent variable (see Figure 1). Three process variables were assessed: 1) self-focused attention, 2) negative social cognitions; and 3) depressed mood.

All variables were measured at Level 1 following the mediation procedure described by Bauer et al. (2006). To incorporate temporal precedence of the process variable (mediator), lagged scores were used, where social anxiety scores at any given assessment point (time *j*) were regressed on the scores on the process variable at the previous assessment point (time *j-1*). Social anxiety scores from the first week of therapy were therefore not included in the analysis due to the absence of prior scores on the process variable. Social anxiety data from all other available sessions were included, as the model incorporated time gaps between assessment points. Models used robust maximum likelihood estimation (MLR). Path *a* (regression of the process variable on the independent variable) and path *c*’ (regression of the dependent variable on the independent variable, in the presence of the process variable) were allowed to vary across participants and were therefore estimated as random, while path *b* (regression of the dependent variable on the process variable) was modelled as a fixed effect. This was done both to limit model complexity, and because the extent of between-subject variability in this relationship was not of primary interest in this study. To prevent the conflation of within- and between-subjects variance, independent and process variables were disaggregated into within- and between-level components via group mean centering. The participant (group) mean-centered scores, and the participant mean scores across all timepoints therefore represented the within and between components of these variables, respectively, and were entered into the model separately (see Hoffart, Borge, & Clark, 2016; Preacher et al., 2010). This approach therefore permits the examination of within-subjects effects, controlling for between-subjects effects.

To further examine the direction of the mediated effect, a series of models were computed which were identical to the models described above apart from the process and outcome variables, which were swapped. These therefore examined the ‘reversed’ relationship, using social anxiety at time *j-1* as the potential mediator, and self-focused attention, negative social cognitions, or depressed mood at time *j* as the dependent variable.

Percent mediation (P_M_) of outcome by the process variable was calculated as an indicator of the strength of any indirect effects following the procedures described in Kenny et al. (2003) and Moscovitch et al. (2005); P_M_ = 100 × [((*ab* + *c*’ + σ*_ab_*) - *c*’) / (*ab* + *c*’ + σ*_ab_*)], where *a*, *b*, and *c*’ represent the respective path coefficients, and σ*_ab_* is the covariance between *a* and *b*. However, as path *b* was specified as fixed, and the covariance between a random and fixed path equals zero, the formula simplifies to P_M_ = 100 × (*ab* / *ab* + *c*’), and the indirect effect to *a* × *b* (see Mörtberg et al., 2015).

Analyses were performed using MPlus version 7.0 (Muthén & Muthén, 1998-2015) and R version 3.4.3 (R Core Team, 2017) using the R package ‘MplusAutomation’ (Hallquist & Wiley, 2018). Inspection of the intraclass correlation coefficients for each model indicated sufficient between-subject variance to justify multilevel analysis (ICC = .43 – .68). Alongside *p*-values, confidence intervals of parameter estimates were reviewed to assess statistical significance.

## Results

Baseline and end of treatment means and standard deviations for Samples 1 and 2 are shown in Table 2. Significant decreases were observed across treatment on all of the measures assessed.

**Table 2:** Baseline and End of Treatment Mean Scores for Samples 1 and 2

Measure	Baseline *M (SD)*	End of treatment *M (SD)*	Test statistic	Pre-post *d*_Cohen_ [95% CI]
Sample 1 (*n* = 185)				
SPWSS (4-item)	21.07 (5.04)	9.41 (7.18)	*t*(183) = 22.23, *p* < .001	1.88 [1.63, 2.12]
SFA	5.30 (1.48)	2.52 (1.75)	*t*(176) = 18.59, *p* < .001	1.72 [1.47, 1.96]
SCQ-c	1.38 (1.22)	-0.91 (1.36)	*t*(182) = 22.77, *p* < .001	1.77 [1.53, 2.01]
BDI	18.32 (11.03)	7.94 (10.07)	*t*(184) = 14.68, *p* < .001	0.98 [0.77, 1.20]
Sample 2 (*n* = 86)				
SPIN	41.52 (12.72)	21.71 (15.67)	*t*(70) = 12.35, *p* < .001	1.39 [1.04, 1.74]
SFA	4.82 (1.80)	2.92 (1.83)	*t*(82) = 9.21, *p* < .001	1.05 [0.73, 1.37]
SCQ-c	1.42 (1.29)	-0.74 (1.34)	*t*(77) = 15.34, *p* < .001	1.64 [1.29, 2.00]
PHQ	11.17 (6.85)	5.21 (6.17)	*t*(83) = 9.59, *p* < .001	0.91 [0.60, 1.23]

*Note.* Baseline (Pre) scores are taken from the initial assessment, or the Session 1 score in cases where no assessment score was available. End of treatment (Post) scores used the last available score. *t* statistics represent paired *t*-tests comparing baseline and end of treatment scores. SPWSS = Social Phobia Weekly Summary Scale; SFA = Self-Focused Attention; SCQ-c = Social Cognitions Questionnaire – composite *z* score; BDI = Beck Depression Inventory. SPIN = Social Phobia Inventory; PHQ = Patient Health Questionnaire. *d*_Cohen_ calculated using the pooled standard deviation as the denominator, calculated as SQRT((*SD*^2^_initial_ + *SD*^2^_post_) / 2) (Van Etten & Taylor, 1998). Confidence intervals for *d*_Cohen_ were calculated using the Hedges and Olkin formula (see Lee, 2016). Cohen (1988) suggested that broadly, effect sizes of 0.2, 0.5, and 0.8 indicated small, medium, and large effects, respectively.

### Sample 1

Results of the MSEM models are shown in Table 3. Significant indirect effect estimates were observed for all three of the process variables assessed, indicating that self-focused attention, negative social cognitions, and depressed mood all mediated the effect of time on social anxiety. The significant and negative path *a* coefficients highlighted that as time in therapy increased, scores on the process variables decreased, with the significant, positive path *b* coefficients indicating that these lower scores predicted lower social anxiety at the following assessment. Inspection of the percent mediation values indicated that negative social cognitions showed the strongest indirect effect.

**Table 3:** Model Results for Sample 1: Unstandardised Path Coefficients, Random Slope Variances, and Indirect Effect Estimates

Parameter	Self-focused attention				Negative social cognitions				Depressed mood			
	Estimate	*SE*	*p*	P_M_	Estimate	*SE*	*p*	P_M_	Estimate	*SE*	*p*	P_M_
*a*	-0.013	0.001	< .001		-0.011	0.001	< .001		-0.035	0.003	< .001	
*b*	0.821	0.100	< .001		1.922	0.162	< .001		0.162	0.034	< .001	
*c’*	-0.039	0.002	< .001		-0.029	0.002	< .001		-0.044	0.003	< .001	
Var*_a_*	< 0.001	< 0.001	< .001		< 0.001	< 0.001	< .001		0.001	< 0.001	< .001	
Var*_c_*_’_	0.001	< 0.001	< .001		< 0.001	< 0.001	< .001		0.001	< 0.001	< .001	
Indirect effect *ab*	-0.010	0.001	< .001	21	-0.021	0.002	< .001	42	-0.006	0.001	< .001	11
Models reversing process variable and outcome												
*a*	-0.053	0.003	< .001		-0.053	0.03	< .001		-0.053	0.003	< .001	
*b*	0.096	0.008	< .001		0.073	0.005	< .001		0.159	0.030	< .001	
*c’*	-0.006	0.001	< .001		-0.005	< 0.001	< .001		-0.022	0.003	< .001	
Var*_a_*	0.001	< 0.001	< .001		0.001	< 0.001	< .001		0.001	< 0.001	< .001	
Var*_c_*_’_	^(see notes)^				^(see notes)^				0.001	< 0.001	< .001	
Indirect effect *ab*	-0.005	0.001	< .001	46	-0.004	< 0.001	< .001	43	-0.008	0.002	< .001	28

*Note. n* = 185. Path *a* represents the effect of time on the process variable. Path *b* represents the effect of the process variable on social anxiety score at the subsequent assessment (with time held constant). Path *c*’ represents the effect of time on social anxiety score controlling for the effect of the process variable. Path *ab* represents the indirect, or mediated, effect. The ‘reversed’ models swap the process and outcome variables. *SE* = standard error, P_M_ = percent mediation (i.e. the percentage of the total effect of time on outcome score that is accounted for by the mediated path *ab*), Var = variance. Due to lack of model convergence when the *c*’ path was specified as random, the reversed models for self-focused attention and negative social cognitions were run using a fixed *c*’ path therefore no variance is given.

The reversed models, which swapped the social anxiety and process variables but retained the time-lag component, were also significant for the three process variables assessed. These findings suggest that lower social anxiety scores were associated with subsequent reduced self-focused attention, reduced negative social cognitions, and improved mood at the following assessment. The percent mediation values for these models were similar across the three variables examined.

### Sample 2

Results of the MSEM models for Sample 2 are shown in Table 4. These models also showed significant indirect effect estimates for all three of the process variables assessed (self-focused attention, negative social cognitions, and depressed mood), indicating that these variables mediated the effect of time on social anxiety as measured by the SPIN. The percent mediation values again indicated that negative social cognitions showed the strongest effect, though the strength of the indirect effect for self-focused attention was weaker in Sample 2 compared to Sample 1. The reversed models were significant for the three process variables assessed, with similar percent mediation values across the three variables, as was observed in Sample 1. Overall, the consistency of model results between the two samples was high, suggesting the Sample 1 findings were replicated in Sample 2.

**Table 4:** Model Results for Sample 2: Unstandardised Path Coefficients, Random Slope Variances, and Indirect Effect Estimates

Parameter	Self-focused attention				Negative social cognitions				Depressed mood			
	Estimate	*SE*	*p*	P_M_	Estimate	*SE*	*p*	P_M_	Estimate	*SE*	*p*	P_M_
*a*	-0.016	0.001	< .001		-0.016	0.001	< .001		-0.036	0.005	< .001	
*b*	0.796	0.250	.001		3.199	0.448	< .001		0.428	0.123	< .001	
*c’*	-0.128	0.011	< .001		-0.092	0.010	< .001		-0.127	0.011	< .001	
Var*_a_*	< 0.001	< 0.001	< .001		< 0.001	< 0.001	< .001		0.001	< 0.001	< .001	
Var*_c_*_’_	0.007	0.001	< .001		0.005	0.001	< .001		0.007	0.001	< .001	
Indirect effect *ab*	-0.013	0.004	.003	9	-0.050	0.008	< .001	35	-0.015	0.005	.002	11
Models reversing process variable and outcome												
*a*	-0.158	0.012	< .001		-0.158	0.012	< .001		-0.159	0.012	< .001	
*b*	0.030	0.007	< .001		0.040	0.005	< .001		0.078	0.021	< .001	
*c’*	-0.010	0.002	< .001		-0.007	0.001	< .001		-0.021	0.005	< .001	
Var*_a_*	0.010	0.002	< .001		0.010	0.002	< .001		0.010	0.002	< .001	
Var*_c_*_’_	< 0.001	< 0.001	< .001		^(see notes)^				0.001	< 0.001	.001	
Indirect effect *ab*	-0.005	0.001	< .001	32	-0.006	0.001	< .001	47	-0.012	0.004	.001	37

*Note. n* = 86. Path *a* represents the effect of time on the process variable. Path *b* represents the effect of the process variable on social anxiety score at the subsequent assessment (with time held constant). Path *c*’ represents the effect of time on social anxiety score controlling for the effect of the process variable. Path *ab* represents the indirect, or mediated, effect. The ‘reversed’ models swap the process and outcome variables. *SE* = standard error, P_M_ = percent mediation (i.e. the percentage of the total effect of time on outcome score that is accounted for by the mediated path *ab*), Var = variance. Due to lack of model convergence when the *c*’ path was specified as random, the reversed model for negative social cognitions was run using a fixed *c*’ path therefore no variance is given.

## Discussion

This study aimed to examine whether self-focused attention, negative social cognitions, and depressed mood were associated with clinical improvement in CT-SAD delivered in a routine clinic setting. Negative social cognitions were supported as a mediator of clinical improvement in Samples 1 and 2, showing the strongest percent mediation values of the three process variables assessed. The results therefore support the Clark and Wells (1995) model that underpins the treatment, and suggest that one of the reasons why people experience less social anxiety as they progress through treatment is that they are experiencing fewer and less-convincing negative thoughts about social situations. These findings are in line with a number of other studies investigating cognitions as a possible process variable driving improvements in social anxiety (Boden et al., 2012; Calamaras et al., 2015; Goldin et al., 2014; Gregory et al., 2018; Hoffart et al., 2012).

Self-focused attention was supported as a mediator of clinical improvement in both Samples 1 and 2, suggesting that successfully shifting towards a more external focus of attention is one reason for the reduction in social anxiety as time in therapy increases. These findings are consistent with the three existing studies of self-focused attention (Hedman et al., 2013; Hoffart et al., 2016; Mörtberg et al., 2015), all of which used the same treatment protocol and found process-outcome effects within RCT datasets using analytic approaches similar to the present study. However, the results from both of the present samples indicated a weaker effect for self-focused attention compared to cognitions. This may indicate a distinction between the RCT context and routine practice, for example in how the self-focus aspects of treatment were implemented. The clinical methods to address self-focused attention were further refined during the audit period, so it is likely that not all participants completed an ‘attention training’ session or had this consistently emphasised during treatment. In contrast, participants in the Hoffart et al. (2016), Hedman et al. (2013), and Mörtberg et al. (2015) studies all completed a specific attention training session, and were supported to practise externally focused attention throughout therapy. It is possible that the lesser emphasis on targeting self-focused attention in the present samples, especially in comparison to targeting cognitions, may help to explain the differences observed in the strength of these effects.

Depressed mood showed significant mediation across Samples 1 and 2, though the percent mediation values indicated a weaker relationship compared to negative social cognitions. The weaker and less consistent effects observed for this variable, which is not part of the theoretical model underpinning CT-SAD, therefore lend some support to the specificity of the effects found for the theoretically-derived process variables.

It is notable that for both Samples 1 and 2, significant ‘reversed’ effects were observed across the three process variables, with similar or greater percent mediation values than in the forward models. This may indicate a cyclical relationship between process and outcome, where changes in negative cognitions and self-focused attention lead to subsequent reductions in social anxiety, and in addition, reductions in social anxiety have a beneficial effect in reducing negative social beliefs and perhaps reducing the perceived need to monitor and focus on yourself in social situations.

### Strengths and Limitations

From a methodological perspective, the use of an additional process variable that is not part of the theoretical model being tested, and statistical methods such as MSEM to account for repeated-measures data and the between- and within-person variance were strengths of the present work and should be considered for future studies in this area. Reversed models are also not implemented consistently and are therefore recommended. While the division of the data into two samples was necessary given the different outcome measures used, it provided a helpful opportunity to assess whether the Sample 1 results would replicate, and the similarity of the results between samples affords increased confidence in the findings. The self-focused attention models may be limited by the use of a two-item mean score to measure this construct. While this measure has been used previously (Hoffart et al., 2016; Mörtberg et al., 2015) future research could usefully develop more nuanced tools to monitor change in this variable over time. It remains possible that other process variables not assessed in the present studies could show strong associations with outcome; for example the use of safety behaviours would be hypothesised as a mediator based on the Clark and Wells (1995) model, but could not be examined here given this was not measured weekly. It is noted also that the present models only examine consecutive timepoints (usually weekly measures), so do not assess process-outcome effects on broader levels, for example delayed or cumulative effects of changes in process variables.

### Conclusion

Overall, the present study found that in routine clinical practice, three process variables (negative social cognitions, self-focused attention, and depressed mood) were associated with subsequent social anxiety outcomes in CT-SAD, with negative social cognitions showing the strongest and most consistent effect. The findings are therefore in line with the Clark and Wells (1995) model that underpins the treatment, and are consistent with RCT-based research findings examining cognitive-behavioural therapies for SAD. Further work examining associations between process variables and clinical outcomes within datasets from routine clinical practice is recommended.
